# Evolution of household availability of regional foods in Amazonas

**DOI:** 10.11606/s1518-8787.2023057004804

**Published:** 2023-09-29

**Authors:** Rita de Cássia de Assunção Monteiro, Eliseu Verly

**Affiliations:** I Universidade do Estado do Amazonas Escola Superior de Ciências da Saúde Manaus AM Brazil Universidade do Estado do Amazonas. Escola Superior de Ciências da Saúde. Manaus, AM, Brazil.; II Universidade do Estado do Rio de Janeiro Instituto de Medicina Social Departamento de Epidemiologia Rio de Janeiro RJ Brazil Universidade do Estado do Rio de Janeiro. Instituto de Medicina Social. Departamento de Epidemiologia. Rio de Janeiro, RJ, Brazil.

**Keywords:** Basic Nutrition, Feeding Behavior, Food Intake, Dietary Surveys

## Abstract

**OBJECTIVE:**

To evaluate the evolution of household availability of regional foods in the state of Amazonas, their distribution according to sociodemographic characteristics, and potential differences when compared to the remaining areas of Brazil.

**METHODS:**

Data on food acquisition for home consumption from the 2002-2003, 2008-2009, and 2017-2018 *Pesquisa de Orçamentos Familiares* (POFs – Consumer Expenditure Surveys) were analyzed, covering, respectively, 48,470, 55,970, and 57,920 households in Brazil, of which 1,075, 1,344, and 1,833 are in Amazonas. Foods were categorized into three groups: cassava and its derivatives, freshwater fish, and regional fruits. The study analyzed the amount of regional food purchased, expressed in relative household caloric share, for the entire area of Amazonas. Additionally, the data was stratified and analyzed according to sociodemographic variables, with differences assessed through the overlapping of 95% confidence intervals.

**FINDINGS:**

The household caloric share of the total regional foods in Amazonas was 22.54% in 2002-2003, 18.18% in 2008-2009, and 6.49% in 2017-2018. Across Brazil, those percentages were much lower in the same period: 3.67%, 3.34%, and 1.82%, respectively. Changes in Amazonas were primarily attributed to the steep drop in the cassava and derivatives group, which decreased from 14.30% in 2002-2003 to 12.74% in 2008-2009 and further declined to 3.09% in 2017-2018. Additionally, there was a gradual decline in household availability of freshwater fish, decreasing from 7.30% in 2002-2003 to 4.85% in 2008-2009 and reaching 2.90% in 2017-2018. Households in rural areas and with lower per capita income presented a higher proportion of calories from total regional foods; this particular stratum also experienced the most significant reductions in their consumption.

**CONCLUSION:**

During the study period, there was a significant decrease in the consumption of regional foods in Amazonas, particularly in lower income households in rural areas. Among them, the family reference person was typically a younger male with a lower educational background.

## INTRODUCTION

Restoring eating habits as a broad construct, encompassing traditional, economically, and environmentally sustainable food consumption, while respecting regional specificities as a means to enhance health, has become deeply ingrained in public food and nutrition policies in the country^1,2^.

From a national standpoint, the process of food transition in recent decades has witnessed substantial dietary composition changes, with traditional staples such as rice, beans, milk, flour, soy oil, and sugar being supplanted by ready meals and industrialized mixes, widely known for their poor nutritional quality^3,4^. However, this transformation does not seem to occur uniformly across all regions of the country.

Although different food availability and consumption profiles linked to the dietary habits of the five Brazilian macro-regions are recognized, these differences have not been thoroughly investigated or analyzed in terms of dietary particularities at the state level^3,5^. For instance, the national trend of a decrease in the consumption of cereals and legumes is not mirrored in Amazonas, where a certain stability of items perceived as foundational in the Brazilian diet, such as rice, beans, and wheat flour^6,7^, is observed. Similarly, a study on dietary behaviors found a prevailing “rice and beans” pattern across all regions of the country, except in the North Region, where the primary diets featured cassava flour, fish, and oilseeds, representing typical regional foods^8^.

Regional food means all that which is regarded as local, native, characteristic, or adapted within the region and is also considered a significant marker of cultural identity. It typically consists of fresh, easily accessible, and affordable products that contribute to sustainability by generating income, jobs, and facilitating a closer connection between production and consumption^9^.

Despite being rooted in the habits and preferences of the population, regional food has been underutilized as evidenced by a few specific studies in school^10,11^ and institutional meals^12,13^.

In Amazonas, food has distinct peculiarities, heavily influenced by Indigenous traditions and boasting a diverse array of natural resources that hold the potential to support a well-balanced diet. However, there is little information concerning food in the state, particularly regarding how the consumption of regional foods has evolved in recent years. Existing local and national publications barely highlight the prominent inclusion of fish and cassava flour in the Amazonian diet^5,14,15^.

Understanding food within its various regional contexts is crucial to ensuring that initiatives aimed at promoting healthy eating align with the specific circumstances and feasibility of each region. With this in mind, our study seeks to assess the trends in household availability of regional foods in Amazonas while examining their distribution across a range of sociodemographic characteristics and exploring potential disparities in comparison to the rest of Brazil.

## METHODS

### Data Source, Sample, and Data Collection

The study utilized data from three editions of the *Pesquisa de Orçamentos Familiares* (POF – Consumer Expenditure Survey), conducted by the Brazilian Institute of Geography and Statistics (IBGE), encompassing the years 2002-2003, 2008-2009, and 2017-2018. POF employed a complex sampling plan using conglomerates in two stages involving census sectors and households to ensure representative results for households across different regions and urban/rural areas of the country, as detailed in its issues^6,7,16^.

The study included data from a total of 48,470 households interviewed in 2002-2003, 55,970 households in 2008-2009, and 57,920 households in 2017-2018 throughout Brazil. Specifically focusing on the state of Amazonas, where 1,075 households were interviewed in 2002-2003, 1,344 in 2008-2009, and 1,833 in 2017-2018. The sampling of sectors for data collection was uniformly distributed across the four quarters, considering the seasonal variations in budget and expenses during the study period^6,7,16^.

### Data Organization, Variables, and Analysis Definition

It was found that Brazilian households purchased approximately 5,400 food and beverage items during a seven-day period in POF 2002-2003, with an increase to around 7,900 items in 2008-2009, and approximately 8,300 items in 2017-2018. Residents of the households or interviewers recorded the details of the items purchased, including the quantities and how they were purchased, in an acquisition book^6,7,16^. The researchers referred to relevant literature sources to identify regional foods specific to Amazonas^9,17^. Based on this information, they were classified into three groups:

- Cassava and derivatives – include various types of cassava, flour, and cassava starch, along with tapioca gum.
- Freshwater fish – encompasses all types of freshwater and unspecified fish.
- Regional fruits – abiu, apricot, acerola, araçá, bacuri, plantain banana, biribá, cocoa, cajarana cherry, carambola, cupuaçu, sugar apple, breadfruit, soursop, guarana, ingá, jambo, genipap, mangaba, murici, pitanga, pitomba, sapote, sapoti, tamarind, taperebá (brazilian apricot), umari, uxi, açaí, bacaba, buriti, brazil nut, inajá, patauá, piquiá nut, pupunha, and tucumã.

A corresponding correction factor was applied, as needed, to determine the edible fraction from the gross quantities of purchased foods^18^. The total amounts of each food item, measured in grams of the edible fraction, were then converted into kilocalories (Kcal) using the Tables of Nutritional Composition of Food Consumed in Brazil^19^. Next, the purchased amounts of each regional food within each group, in kilocalories, were summed up per household, considering the data aggregated over seven consecutive days, and then divided by the collection period to match it to the information from a single day of effective acquisition. A variable describing the total calories from regional foods purchased by households was obtained by summing the total calories of the three groups listed.

The caloric contribution of regional foods for each household was calculated as a percentage, representing the ratio between the calories of regional items and the sum of the calories from all food purchased by the household, multiplied by 100. The amount of regional food purchased was expressed as the relative household caloric participation (indicated as a percentage) for both the total of regional foods and each of the three specific food groups.

The acquisition of regional foods in Amazonas was analyzed in its entirety and further examined based on sociodemographic variables, including household situation (urban and rural), per capita family monthly income each quarter (with the lowest income represented by the 1^st^ quarter), gender, age, and education of the reference person in the family. Additionally, regional food procurement was analyzed for the remainder of Brazil as a whole, aiming to make comparisons with the estimates for the state of Amazonas.

The average caloric contribution of regional foods between the categories of variables used in this study was compared using 95% confidence intervals. Differences were deemed statistically significant when confidence intervals did not overlap.

All analyses were performed using Stata software, version 13.0 (StataCorp L.P., College Station, Texas, United States), employing the *survey* module, which takes the complex sampling design of the POF into account.

## RESULTS

The analysis of the first two editions of the POF revealed that the total number of calories available at home from regional foods accounted for 22.54% in 2002-2003 and 18.18% in 2008-2009 in Amazonas. In contrast, in the rest of Brazil, the relative caloric participation of regional foods was much smaller during the same period, amounting to 3.67% and 3.34%, respectively. However, the subsequent edition of the POF in 2017-2018 showed that this difference became less pronounced, with regional foods contributing to only 6.49% of total calories in Amazonas and 1.82% in the rest of the country. The magnitude of the difference between the share of regional foods in Amazonas and the rest of the country was halved from 2002-2003 to 2017-2018 (Table 1).

**Table 1:** Table 1 presents the mean values (95%CI) of the relative caloric share of regional foods in total household energy availability in Amazonas and the rest of Brazil for POFs 2002-2003, 2008-2009, and 2017-2018.

Food Groups/Locations	2002-2003	2008-2009	2017-2018
Cassava and derivatives			
Amazonas	14.30 (13.13–15.48)	12.74 (11.51–13.97)	3.08 (2.53–3.62)^b^
Across Brazil	3.20 (3.10–3.30)	2.63 (2.52–2.73)^a^	1.16 (1.09–1.22)^b^
Freshwater fish			
Amazonas	7.30 (6.43–8.17)	4.85 (4.10–5.60)^a^	2.90 (2.06–3.74)^b^
Across Brazil	0.19 (0.17–0.20)	0.36 (0.32–0.40)^a^	0.26 (0.22–0.30)^b^
Regional fruits			
Amazonas	0.94 (0.57–1.31)	0.53 (0.40–0.67)	0.49 (0.33–0.66)
Across Brazil	0.22 (0.20–0.24)	0.27 (0.24–0.30)	0.29 (0.25–0.33)
Total regional foods			
Amazonas	22.54 (21.18–23.89)	18.13 (16.42–19.84)^a^	6.47 (5.38–7.56)^b^
Across Brazil	3.60 (3.50–3.71)	3.26 (3.14–3.38)^a^	1.70 (1.61–1.79)^b^

^a^ statistically significant difference between POFs 2002-2003 and 2008-2009.

^b^ statistically significant difference between POFs 2008-2009 and 2017-2018.

In Amazonas, these changes were primarily driven by a significant cut in the cassava and derivatives group, which dropped from 14.30% in 2002-2003 to 3.09% in 2017-2018. Another notable trend was the gradual decline in household availability of freshwater fish, decreasing from 7.30% (2002-2003) to 4.85% (2008-2009) and 2.90% (2017-2018). In contrast, the participation of the freshwater fish group across Brazil fluctuated from 0.19% in 2002-2003 to 0.36% in 2008-2009 and 0.26% in 2017-2018.

The group of regional fruits had a relatively small share in the total calories available in households in Amazonas and showed a slight decrease throughout the study period, remaining below 1.00% in all editions of the research (Table 1).

The examination of total regional food purchases by household situation revealed that in the rural environment, the average relative caloric participation exceeded that of the urban environment by approximately three times in 2002-2003, two times in 2008-2009, and four times in 2017-2018. This difference was statistically significant in all periods and was primarily driven by the freshwater fish group. In 2002-2003, nearly half (47.09%) of the total calories available in rural households were derived from regional foods. However, this share decreased to about one-third (33.61%) in 2008-2009 and further reduced to less than one-fifth (18.48%) in 2017-2018 (Table 2).

**Table 2:** Mean (95%CI) of the relative caloric share of household availability of regional foods in Amazonas by household situation in POFs 2002-2003, 2008-2009, and 2017-2018.

Household situation	2002-2003	2008-2009	2017-2018
Cassava and derivatives			
Urban	11.58 (10.40–12.75)	10.54 (9.52–11.57)	2.72 (2.16–3.27)^b^
Rural	24.82 (21.25–28.40)	21.84 (18.12–25.57)	5.31 (3.59–7.02)^b^
Freshwater fish			
Urban	3.63 (3.13–4.13)	3.50 (2.97–4.03)	1.45 (0.99–1.92)^b^
Rural	21.46 (17.92–24.99)	10.45 (8.00–12.90)^a^	11.92 (5.21–18.62)
Regional fruits			
Urban	0.97 (0.55–1.39)	0.34 (0.21–0.48)^a^	0.37 (0.24–0.51)
Rural	0.81 (0.06–1.55)	1.32 (0.79–1.85)	1.25 (0.35–2.16)
Total regional foods			
Urban	16.18 (14.85–17.51)^c^	14.39 (13.17–15.62)^c^	4.54 (3.76–5.33)^bc^
Rural	47.09 (43.12–51.05)^c^	33.61 (28.23–38.99)^ac^	18.48 (10.84–26.12)^bc^

^a^ statistically significant difference between POFs 2002-2003 and 2008-2009.

^b^ statistically significant difference between POFs 2008-2009 and 2017-2018.

^c^ statistically significant difference in total regional foods across household status.

Furthermore, when analyzing the data by household location, it was observed that the cassava and derivatives group experienced a decrease in both urban and rural areas, but the decline was more pronounced in rural areas, particularly in the last period. In 2017-2018, the share of the group in rural households dropped to 5.31% compared to 21.84% in 2008-2009. The freshwater fish group displayed a significant reduction in the urban environment only from 2008-2009 (3.50%) to 2017-2018 (1.45%). However, in rural areas, the decline in this group was more prominent in the first period, decreasing from 21.46% in 2002-2003 to 10.45% in 2008-2009, with a slight increase in 2017-2018 (11.92%), although not statistically significant. On the other hand, the regional fruits group showed a reduction solely in the urban area, between the first and second surveys, dropping from 0.97% to 0.34%. In rural areas, regional fruits showed a slight increase, although not statistically significant (Table 2).

In the three editions of the POF, households with lower *per capita* income tended to have a greater relative caloric share of freshwater fish, cassava, and derivatives, as well as total regional food. However, there was no relationship between regional fruits and yields at any time (Table 3).

**Table 3:** Mean (95%CI) of the relative caloric share of household availability of regional foods in Amazonas by per capita income quarter in POFs 2002-2003, 2008-2009, and 2017-2018.

Per capita household income each quarter	2002-2003	2008-2009	2017-2018
Cassava and derivatives			
1^st^ quarter	21.60 (18.80–24.40)	16.81 (14.23–19.40)	3.22 (1.93–4.51)^b^
2^nd^ quarter	15.91 (13.25–18.58)	12.90 (11.08–14.71)	3.40 (2.48–4.32)^b^
3^rd^ quarter	11.01 (8.93–13.08)	11.88 (9.82–13.94)	2.68 (1.77–3.60)^b^
4^th^ quarter	7.15 (5.52–8.77)	10.02 (8.37–11.67)	3.01 (1.90–4.13)^b^
Freshwater fish			
1^st^ quarter	8.97 (7.22–10.71)	6.24 (4.38–8.10)	4.32 (1.89–6.74)
2^nd^ quarter	8.25 (6.06–10.44)	4.90 (3.83–5.97)^a^	3.08 (1.89–4.27)
3^rd^ quarter	6.74 (4.73–0.75)	4.27 (3.30–5.25)	2.24 (1.35–3.14)^b^
4^th^ quarter	4.79 (3.11–6.47)	4.19 (3.28–5.11)	2.13 (1.04–3.21)^b^
Regional fruits			
1^st^ quarter	0.74 (0.34–1.14)	0.58 (0.30–0.87)	0.62 (0.28–0.97)
2^nd^ quarter	1.56 (0.47–2.66)	0.76 (0.44–1.07)	0.19 (0.10–0.28)^b^
3^rd^ quarter	0.45 (0.16–0.73)	0.47 (0.13–0.81)	0.41 (0.09–0.74)
4^th^ quarter	1.03 (0.11–1.96)	0.34 (0.19–0.50)	0.75 (0.38–1.12)
Total regional foods			
1^st^ quarter	31.31 (28.00–34.62)	23.64 (19.80–27.48)^a^	8.16 (5.03–11.29)^b^
2^nd^ quarter	25.73 (22.49–28.98)	18.56 (16.42–20.71)^a^	6.67 (5.13–8.22)^b^
3^rd^ quarter	18.19 (15.29–21.09)	16.75 (14.08–19.43)	5.34 (4.08–6.60)^b^
4^th^ quarter	12.97 (10.52–15.42)	14.62 (12.67–16.57)	5.89 (4.24–7.54)^b^

^a^ statistically significant difference between POFs 2002-2003 and 2008-2009.

^b^ statistically significant difference between POFs 2008-2009 and 2017-2018.

In the cassava and derivatives group, there was a significant reduction in all income classes, but only from 2008-2009 to 2017-2018. In the first interval between surveys, the freshwater fish group had a significant decline only in the 2^nd^ quarter, from 8.25% (2002-2003) to 4.90% (2008-2009). In 2017-2018, the decline of the group reached the income strata of the 3^rd^ and 4^th^ quarters. The reduction in household purchases of regional fruits between 2008-2009 and 2017-2018 stands out only in the 4^th^ quarter, dropping from 0.76% to 0.19%. For total regional foods, a statistically significant reduction was observed in the lowest income quarters from 2002-2003 to 2008-2009 and in all income strata in the following period (Table 3).

When analyzing the household availability of total regional foods in Amazonas based on the characteristics of the reference person in the family, a consistent trend was observed across all surveyed periods. There was a tendency towards greater relative caloric participation of regional foods when the reference person was an older male with a lower educational background. Regarding the changes from 2002-2003 to 2008-2009, it was evident that the decline in relative caloric participation was significant for specific groups. The decrease was most significant among males (from 24.42% to 19.37%), individuals up to 39 years old (from 22.19% to 16.61%), and those who attended 0-4 years of schooling (from 32.44% to 24.51%). However, POFs from 2008-2009 to 2017-2018 did not follow the same trend. The reduction in relative caloric participation of total regional food occurred in all analyzed subgroups, irrespective of gender, age group, and education level of the household’s reference person (Table 4).

**Table 4:** Mean (95%CI) of the relative caloric share of household availability of total regional foods in Amazonas according to characteristics of the household’s reference person in POFs 2002-2003, 2008-2009, and 2017-2018.

Sociodemographic variables	2002--2003	2008-2009	2017-2018
Gender of the household's reference person			
Female	17.34 (14.67–20.01)	16.16 (14.59–17.73)	5.10 (4.06–6.15)^b^
Male	24.42 (22.73–26.12)	19.34 (17.07–21.60)^a^	7.47 (5.88–9.06)^b^
Age range of the household's reference person (years)			
Up to 39	22.19 (19.97–24.42)	16.49 (14.47–18.50)^a^	5.09 (3.69–6.48)^b^
40-59	21.57 (19.30–28.83)	17.92 (15.88–19.96)	6.46 (5.13–7.78)^b^
≥ 60	26.22 (21.33–31.10)	22.73 (19.75–25.71)	9.32 (6.57–12.07)^b^
Education of the household's reference person (years)			
20-4	32.44 (29.83–35.05)	24.50 (21.50–27.50)^a^	12.11 (8.17–16.05)^b^
5-8	17.51 (14.99–20.02)	19.34 (16.56–22.12)	6.61 (5.27–7.96)^b^
> 8	12.57 (10.72–14.43)	12.79 (11.46–14.12)	4.87 (3.87–5.87)^b^

^a^ statistically significant difference between POFs 2002-2003 and 2008-2009.

^b^ statistically significant difference between POFs 2008-2009 and 2017-2018.

## DISCUSSION

The analysis of the household share of regional foods in Amazonas over the 15-year period covered by the three POFs has revealed a shifting scenario in the state’s food framework. The comparison between Amazonas and the rest of the country highlights the clear significance that regional foods once held in the local diet. However, the current results evidence that Amazonas is experiencing losses of its distinctive food characteristics, which previously set the state apart from the national context. This trend is consistent with a recent study based on POF data, which found low household availability of regional foods across the country’s macro-regions, signaling a loss of regionality and a downward trend for these items in the Brazilian diet, followed by stagnation, between 2002 and 2018^20^.

Besides the possible effect of such changes, this transformation in food consumption patterns raises concerns about the potential impact on food cultural identity in Amazonas. A significant share of the calories available in households, particularly in rural and lower-income strata, were contributed by cassava and its derivatives, as well as freshwater fish—foods classified as either *in natura* or minimally processed. The traditional food profile in Amazonas, which consisted of fresh foods and culinary preparations, aligned with the recommendations of the Food Guide for the Brazilian population^21^. However, as regional food groups experienced reduced acquisition, there may be inadequate substitutions occurring, impacting food quality. This mirrors the national trend of a progressive shift away from fresh and minimally processed foods, with processed and ultra-processed foods gaining prominence, a pattern also observed in the North Region^22^.

Furthermore, cassava and its derivatives, particularly cassava flour, play a core role in the local food routine and hold cultural significance inherited from the region’s native Indigenous culture^9^. Cassava flour is a valuable source of energy and complex carbohydrates, with a high fiber content (6.5g per 100g of the product) exceeding that of polished rice (1.6g), and brown rice (2.8g). It also contains appreciable amounts of pyridoxine, manganese, magnesium, iron, calcium, and zinc^18^. The potential physiological benefits of cassava flour, attributed to its fiber and resistant starch content, have led to its exploration as a promising functional food^23^.

Fish, in turn, is one of the most abundant natural resources in the region, and its decrease in household purchases is increasingly negative. Besides holding meaning for the Amazonian population that goes beyond the food issue, with extreme cultural and socioeconomic relevance^24,25^, this food is also sought after for its well-known nutritional quality. Scientific evidence consistently supports fish as an excellent alternative for adequate protein intake due to the high amount of essential fatty acids and micronutrients, making it a regularly recommended component of a balanced diet^26^.

However, the reduction in the share of regional foods in rural areas, particularly in Amazonas, is influenced by the seasonality of rivers (periods of flooding, high tides, ebbs, and droughts) directly interferes with fishing activity and local food production. Communities may need to search for alternative sources of nutrients during certain periods of the year when fishing is not as abundant. The last decade has seen notable occurrences of major floods (2009, 2012, and 2015) and severe droughts (2005, 2010, and 2016) in the region, attributed to climate changes, which further exacerbate the challenges faced by rural communities in maintaining access to traditional food sources^27^. The need for daily adaptations due to these environmental fluctuations, combined with financial vulnerability and the allure of modern food choices, may be contributing to the incorporation of new items into families’ eating habits in a more permanent manner.

Qualitative analyses of food transformations within the urban network of Alto Solimões, southwest of Amazonas, have revealed a strong presence of industrialized frozen chicken in the municipalities, partly due to economic reasons^28^.

Similarly, research conducted with low-income families within the Metropolitan Area of Manaus showed that, while fish was the preferred source of animal protein for respondents (41%), chicken was the most frequent food on their tables (69%), regardless of it hardly being their favorite (3%), likely due to income limitations^29^. In fact, the study that sought to assess the panorama of fish consumption by the Brazilian population identified the North region as the only one in which the population has a preference for fish in their meals^30^. In fact, in 2008-2009, chicken accounted for 7.1% of the calories available in households in Amazonas, surpassing fish and becoming the main source of protein in the Amazonian diet^7^.

Considering these dynamics, it is paramount to ensure access to food of proper quality and in sufficient quantity, promoting health-conscious practices that respect biodiversity. The economic and ecological dimensions of food production, marketing, and consumption must be harmonized with socio-environmental sustainability^1^.

Fishing and the cultivation of cassava play crucial roles in the primary sector of Amazonas, generating employment and income for various socioeconomic levels. Particularly for populations living along riverbanks, fishing provides a means of livelihood and helps anchor them to their place of origin. However, fishing in the region still requires improvements in handling, processing, conservation, waste management, marketing, and logistics processes^30^.

Likewise, the cultivation of cassava in the state primarily involves artisanal and family production. It represents an activity that demands minimal investment and simple processing to yield by-products such as flour and starches, which are easily preservable and marketable. Recent local initiatives have aimed to enhance the value of cassava flour by ensuring its origin, environmental sustainability, and support for traditional populations, thus adding value to the regional product and encouraging its consumption^31^.

Despite the strong incentives for cassava flour production, further investments in research and proper management of the flour activity are still needed to promote greater quality control, professionalize the workforce, and enhance the sector’s competitiveness, thereby stimulating the families whose livelihoods are dependent on it. Consequently, fishing activities and the production of cassava flour in Amazonas demand greater attention from official bodies to ensure that they are conducted with social and environmental responsibility, thereby contributing to food security in the region.

One limitation of the study is certainly the omission of food consumption outside of the household. However, the North region has demonstrated the lowest percentages of expenses on food in this context: 19.1% in 2002-2003, 21.4% in 2008-2008, and the same level in 2017-2018, compared to 24.1%, 31.1%, and 32.8% across Brazil, respectively, during the three POF periods^16^. Additionally, research on food consumption outside of the household has revealed that this practice has been more prevalent in urban areas, among younger individuals and those with higher incomes, unlike our study, where the greatest reduction in regional foods occurred in rural areas and among lower-income groups^32^.

Furthermore, part of the observed differences between groups may be attributed to seasonality. The primary initiation units were randomly distributed across the four quarters of the year, ensuring the representation of economic strata in the selected households. However, it does not guarantee an equal distribution of groups, particularly in terms of age and sex, across quarters. In this scenario, the comparison between these categories may be partially distorted.

A notable strength of the study lies in its pioneering analysis of the POF data, with a focus on the purchase of foods endemic to a state. Given Brazil’s diverse food, socioeconomic, and cultural contexts, it is relevant to analyze whether other locations are also experiencing a similar phenomenon of reduced participation of regional items in the diet. Identifying traditional foods that offer incentives in production, reception, and consumption can serve as a relevant initial stage in the process of promoting healthy eating in a manner that aligns with the local culture.

The analysis of the evolution of household availability of regional foods in Amazonas, presumed based on the POFs of 2002-2003, 2008-2009, and 2017-2018, made it possible to comprehend their food characteristics simultaneously, suggesting that the food transition at the local level may not be occurring in the same way as in the framework across the country. There was a significant reduction in the presence of regional foods in the three analyzed periods, primarily affecting households in the rural area and with lower income, as well as families whose reference person was a younger male, with a lower educational background.
