# Supplementary material for: Evolution of household availability of regional foods in Amazonas
Source: Rev Saude Publica. 2023 Sep 29;57:69. doi: 10.11606/s1518-8787.2023057004804 (PMC10547386; doi:10.11606/s1518-8787.2023057004804)
Supplement: Translation

# Translation: Evolução da disponibilidade domiciliar de alimentos regionais no Amazonas

**Authors:** Rita de Cássia de Assunção Monteiro, Eliseu Verly

**Affiliations:** I Brasil Universidade do Estado do Amazonas. Escola Superior de Ciências da Saúde. Manaus, AM, Brasil.; II Brasil Universidade do Estado do Rio de Janeiro. Instituto de Medicina Social. Departamento de Epidemiologia. Rio de Janeiro, RJ, Brasil.

**Keywords:** Alimentação Básica, Comportamento Alimentar, Ingestão de Alimentos, Inquéritos sobre Dietas

## Abstract

**OBJETIVO:**

Avaliar a evolução da disponibilidade domiciliar de alimentos regionais no Amazonas, sua distribuição segundo características sociodemográficas e potenciais diferenças em relação ao restante do Brasil.

**MÉTODOS:**

Foram analisados dados de aquisição de alimentos para consumo domiciliar das Pesquisas de Orçamentos Familiares (POFs) de 2002–2003, 2008–2009 e 2017–2018, que estudaram, respectivamente, 48.470, 55.970 e 57.920 domicílios no Brasil, dos quais 1,075, 1.344 e 1.833 estão no Amazonas. Os alimentos foram reunidos em três grupos: macaxeira e derivados, peixes de água doce, e frutos regionais. A quantidade de alimento regional adquirido, expressa em participação calórica relativa domiciliar, foi analisada para o Amazonas como um todo e segundo variáveis sociodemográficas (diferenças avaliadas pela sobreposição dos intervalos de confiança de 95%).

**RESULTADOS:**

A participação calórica domiciliar do total de alimentos regionais no Amazonas foi de 22,54% em 2002-2003, 18,18% em 2008-2009 e 6,49% em 2017-2018. No Brasil, estes percentuais foram bem menores no mesmo período: 3,67%, 3,34% e 1,82%, respectivamente. As mudanças no Amazonas ocorreram, principalmente, pela drástica redução do grupo de macaxeira e derivados (de 14,30% em 2002–2003 para 12,74% em 2008-2009 e 3,09% em 2017–2018) e pelo declínio gradativo da disponibilidade domiciliar de peixes de água doce (de 7,30% em 2002–2003 para 4,85% 2008–2009 e 2,90% em 2017–2018). Domicílios do meio rural e com menor renda per capita tiveram maior participação calórica do total de alimentos regionais, estratos que também tiveram as maiores reduções.

**CONCLUSÃO:**

Houve redução significativa da presença de alimentos regionais no Amazonas no período estudado, atingindo principalmente os domicílios da zona rural e com menor renda, cuja pessoa de referência da família era do sexo masculino, mais jovem e com menor escolaridade.

## INTRODUÇÃO

O resgate da alimentação como um constructo amplo, que inclui o consumo alimentar tradicional, econômico e ambientalmente sustentável, e que respeite as especificidades regionais como estratégia para a melhoria da saúde, tem permeado as políticas públicas de alimentação e nutrição no país^1,2^.

Numa perspectiva nacional, o processo de transição alimentar nas últimas décadas tem sido marcado por significativas modificações na composição dos cardápios, com a substituição de itens tradicionais como o arroz, feijão, leite, farinhas, óleo de soja e açúcar por refeições prontas e misturas industrializadas, essas últimas caracterizadas pela baixa qualidade nutricional^3,4^. Entretanto, este processo parece não ocorrer de forma homogênea em todas as regiões do país.

Apesar de reconhecer a existência de perfis diferenciados de disponibilidade e de consumo de alimentos relacionados aos hábitos das cinco macrorregiões brasileiras, essas diferenças não têm sido investigadas de forma mais detida, ou as análises não exploram particularidades alimentares por estado^3,5^. Por exemplo, a tendência nacional observada de redução da participação de cereais e leguminosas não se repete no Amazonas, onde se percebe certa estabilidade dos itens considerados básicos na dieta brasileira, como arroz, feijão e farinha de trigo^6,7^. Da mesma forma, estudo de padrões dietéticos encontrou predominância de um padrão denominado “arroz com feijão” em todas as regiões do país, exceto na Região Norte, onde o primeiro padrão foi caracterizado por farinha de mandioca, peixe e oleaginosos, alimentos tipicamente regionais^8^.

Alimento regional é aquele considerado local, nativo, próprio, característico da região ou adaptado, sendo também apontado como um importante marcador da identidade cultural. Geralmente é um produto fresco, de fácil acesso, baixo custo e que contribui para a sustentabilidade, devido à geração de renda, de empregos e da aproximação entre produção e consumo^9^.

Embora o alimento regional constitua, *a priori*, o hábito e a preferência da população, sua subutilização tem sido registrada por alguns poucos estudos pontuais na merenda escolar^10,11^ e na alimentação institucional^12,13^.

No Amazonas a alimentação possui peculiaridades, com forte influência indígena, e dispõe de recursos naturais diversificados, potencialmente capazes de viabilizar uma dieta equilibrada. Entretanto, há pouca informação sobre a alimentação no estado, em particular como se deu a evolução da participação de alimentos regionais nos últimos anos. As publicações locais e nacionais apenas evidenciam a relevante presença do peixe e da farinha de mandioca na dieta amazonense^5,14,15^.

Compreender a alimentação em seus diferentes contextos regionais é essencial para assegurar que as ações de promoção de alimentação saudável sejam consistentes e factíveis. Nesse sentido, o presente trabalho teve como objetivo avaliar a evolução da disponibilidade domiciliar de alimentos regionais no Amazonas, sua distribuição segundo características sociodemográficas, e potenciais diferenças em relação ao restante do Brasil.

## MÉTODOS

### Fonte de Dados, Amostra e Coleta de Dados

O estudo utilizou dados das três últimas edições da Pesquisa de Orçamentos Familiares (POF), 2002–2003, 2008–2009 e 2017–2018, conduzidas pelo Instituto Brasileiro de Geografia e Estatística (IBGE). A POF utiliza plano amostral complexo, por conglomerados em dois estágios, com sorteio de setores censitários e de domicílios, sendo representativa do conjunto de domicílios do país por região, situação urbana e rural, conforme descrito em suas publicações^6,7,16^.

Foram utilizados dados referentes ao total de domicílios entrevistados em 2002–2003, 2008–2009 e 2017–2018 no Brasil (48.470, 55.970 e 57.920, respectivamente), dos quais, 1.075, 1.344 e 1.833 pertencentes ao estado do Amazonas. Os setores foram distribuídos ao longo dos quatro trimestres da coleta de dados de maneira uniforme, contemplando as sazonalidades do orçamento e das despesas no período^6,7,16^.

### Organização dos Dados, Definição das Variáveis e Análises

Foi relatada pelos domicílios brasileiros a aquisição de aproximadamente 5.400 (POF 2002-2003), 7.900 (POF 2008–2009) e 8.300 (POF 2017–2018) itens de alimentos e bebidas durante um período de sete dias consecutivos. Os itens adquiridos, a quantidade e a forma de aquisição foram registrados pelos moradores do domicílio ou pelo entrevistador em uma caderneta de aquisição^6,7,16^. A partir dessa lista os alimentos foram classificados como regionais do Amazonas, com base na literatura específica^9,17^, sendo reunidos em três grupos:

- Macaxeira e derivados – macaxeira, farinha e fécula de mandioca de diferentes tipos, incluindo goma de tapioca.
- Peixes de água doce – todos os peixes de água doce e peixe não especificado;
- Frutos regionais – abiu, abricó, acerola, araçá, bacuri, banana pacovã, biribá, cacau, cajarana, carambola, cupuaçu, fruta de conde, fruta pão, graviola, guaraná, ingá, jambo, jenipapo, mangaba, murici, pitanga, pitomba, sapota, sapoti, tamarindo, taperebá, umari, uxi, açaí, bacaba, buriti, castanha do Brasil, inajá, patauá, piquiá, pupunha e tucumã.

Para obtenção da fração comestível a partir das quantidades brutas dos alimentos adquiridos, foi aplicado, quando apropriado, o fator de correção correspondente^18^. As quantidades totais de cada item, em gramas da fração comestível, foram convertidas em quilocalorias (Kcal) com auxílio das Tabelas de Composição Nutricional dos Alimentos Consumidos no Brasil^19^. Em seguida, as quantidades adquiridas de cada um dos alimentos regionais dentro de cada grupo, em Kcal, foram somadas por domicílio. A informação de aquisição, agregada em sete dias consecutivos, foi dividida pelo seu período de coleta, equiparando-a ao período de informação de um dia de aquisição efetiva. Uma variável descrevendo o total de calorias a partir dos alimentos regionais adquiridos pelos domicílios foi obtida a partir do somatório do total calórico dos três grupos listados acima.

A contribuição calórica dos alimentos regionais (em porcentagem) foi calculada para cada domicílio como a razão entre as calorias dos itens regionais e o somatório das calorias do total de alimentos adquiridos pelo domicílio, multiplicado por 100. A quantidade de alimento regional adquirido foi expressa em participação calórica relativa domiciliar (apresentada em porcentagem) para o total de alimentos regionais e para cada um dos três grupos de alimentos definidos.

A aquisição de alimentos regionais foi analisada para o Amazonas como um todo e segundo as variáveis sociodemográficas: situação do domicílio (urbano e rural); quartos de renda mensal familiar per capita (em Reais, sendo a menor renda representada pelo 1º quarto); sexo, idade e escolaridade da pessoa de referência da família. Adicionalmente, a aquisição de alimentos regionais foi analisada para o restante do Brasil como um todo, com o objetivo de a comparar com as estimativas do Amazonas.

A comparação das médias de contribuição calórica dos alimentos regionais entre as categorias das variáveis utilizadas neste estudo se deu pelos intervalos de confiança de 95%. Foram consideradas diferenças estatisticamente significativas quando os intervalos de confiança não se sobrepuseram.

As análises foram realizadas utilizando o software Stata, versão 13.0 (StataCorp L.P., College Station, Texas, United States) no módulo *survey*, que considera a amostragem complexa da POF.

## RESULTADOS

Nas duas primeiras edições da POF, o total de calorias disponíveis no domicílio com alimentos regionais alcançou, no Amazonas, 22,54%, em 2002–2003, e 18,18% em 2008-2009, enquanto, no restante do Brasil, apresentaram uma participação calórica relativa bem menor no mesmo período, 3,67% e 3,34%, respectivamente. Contudo, em 2017–2018 essa diferença deixou de ser tão evidente, com o total de alimentos regionais correspondendo somente a 6,49% das calorias totais no Amazonas e 1,82% no restante do país. Com isso, a magnitude da diferença entre a participação de alimentos regionais no estado selecionado e no restante do país foi reduzida pela metade de 2002–2003 para 2017–2018 (Tabela 1).

**Tabela 1:** Média (IC95%) da participação calórica relativa de alimentos regionais na disponibilidade domiciliar total de energia no Amazonas e no restante do Brasil nas POFs 2002–2003, 2008–2009 e 2017–2018.

Grupos de alimentos/Locais	2002-2003	2008-2009	2017-2018
Macaxeira e derivados			
Amazonas	14,30 (13,13–15,48)	12,74 (11,51–13,97)	3,08 (2,53–3,62)^b^
Restante do Brasil	3,20 (3,10–3,30)	2,63 (2,52–2,73)^a^	1,16 (1,09–1,22)^b^
Peixes de água doce			
Amazonas	7,30 (6,43–8,17)	4,85 (4,10–5,60)^a^	2,90 (2,06–3,74)^b^
Restante do Brasil	0,19 (0,17–0,20)	0,36 (0,32–0,40)^a^	0,26 (0,22–0,30)^b^
Frutos regionais			
Amazonas	0,94 (0,57–1,31)	0,53 (0,40–0,67)	0,49 (0,33–0,66)
Restante do Brasil	0,22 (0,20–0,24)	0,27 (0,24–0,30)	0,29 (0,25–0,33)
Total de alimentos regionais			
Amazonas	22,54 (21,18–23,89)	18,13 (16,42–19,84)^a^	6,47 (5,38–7,56)^b^
Restante do Brasil	3,60 (3,50–3,71)	3,26 (3,14–3,38)^a^	1,70 (1,61–1,79)^b^

^a^ diferença estatisticamente significativa entre a POF 2002–2003 e a POF 2008–2009.

^b^ diferença estatisticamente significativa entre a POF 2008–2009 e a POF 2017–2018.

As mudanças observadas no Amazonas ocorreram, principalmente, pela drástica redução do grupo de macaxeira e derivados, que, de 14,30%, em 2002-2003, passou para 3,09% em 2017-2018. Outro destaque foi o declínio gradativo da disponibilidade domiciliar de peixes de água doce, de 7,30% (2002–2003) para 4,85% (2008–2009) e 2,90% (2017–2018). Em oposição, no restante do Brasil, a participação desse grupo oscilou de 0,19%, em 2002–2003, para 0,36%, em 2008–2009, e 0,26% em 2017–2018 (Tabela 1).

O grupo dos frutos regionais teve participação relativamente pequena no total de calorias disponíveis nos domicílios do Amazonas. Apresentou, no período estudado, decréscimo, embora não significativo, mantendo-se abaixo de 1,00% em todas as edições da pesquisa (Tabela 1).

A verificação da aquisição do total de alimentos regionais por situação do domicílio demonstrou que, no meio rural, a participação média calórica relativa excedeu em cerca de três (2002–2003), duas (2008–2009) e quatro vezes (2017–2018) a do meio urbano. Essa diferença se mostrou significativa em todos os momentos e ocorreu, principalmente, devido ao grupo de peixes de água doce. Em 2002–2003, quase metade (47,09%) das calorias totais disponíveis nos domicílios do meio rural eram contempladas por alimentos regionais, participação que reduziu para cerca de um terço (33,61%) e para menos de um quinto (18,48%), nos anos de 2008–2009 e 2017–2018, respectivamente (Tabela 2).

**Tabela 2:** Média (IC95%) da participação calórica relativa da disponibilidade domiciliar de alimentos regionais no Amazonas por situação do domicílio nas POFs 2002–2003, 2008–2009 e 2017–2018.

Situação do domicílio	2002–2003	2008–2009	2017–2018
Macaxeira e derivados			
Urbano	11,58 (10,40–12,75)	10,54 (9,52–11,57)	2,72 (2,16–3,27)^b^
Rural	24,82 (21,25–28,40)	21,84 (18,12–25,57)	5,31 (3,59–7,02)^b^
Peixes de água doce			
Urbano	3,63 (3,13–4,13)	3,50 (2,97–4,03)	1,45 (0,99–1,92)^b^
Rural	21,46 (17,92–24,99)	10,45 (8,00–12,90)^a^	11,92 (5,21–18,62)
Frutos regionais			
Urbano	0,97 (0,55–1,39)	0,34 (0,21–0,48)^a^	0,37 (0,24–0,51)
Rural	0,81 (0,06–1,55)	1,32 (0,79–1,85)	1,25 (0,35–2,16)
Total de alimentos regionais			
Urbano	16,18 (14,85–17,51)^c^	14,39 (13,17–15,62)^c^	4,54 (3,76–5,33)^bc^
Rural	47,09 (43,12–51,05)^c^	33,61 (28,23–38,99)^ac^	18,48 (10,84–26,12)^bc^

^a^ diferença estatisticamente significativa entre a POF 2002–2003 e a POF 2008–2009.

^b^ diferença estatisticamente significativa entre a POF 2008–2009 e a POF 2017–2018.

^c^ diferença estatisticamente significativa no total de alimentos regionais entre situação do domicílio.

Ademais, a análise por localização do domicílio indicou que o grupo de macaxeira e derivados teve decréscimo em ambos os estratos, porém de forma mais acentuada no meio rural, em especial no último período, quando reduziu de 21,84%, em 2008–2009, para 5,31%, em 2017-2018. Já o grupo de peixes de água doce teve redução significativa no meio urbano somente de 2008–2009 (3,50%) para 2017–2018 (1,45%). Contudo, no meio rural, o declínio desse grupo foi mais acentuado apenas no primeiro período: de 21,46%, em 2002–2003, para 10,45% em 2008-2008, havendo, inclusive, um leve aumento em 2017–2018 (11,92%), embora não significativo. Já o grupo de frutos regionais apresentou redução apenas na área urbana, e entre o primeiro e o segundo inquérito, passando de 0,97% para 0,34%. Na zona rural os frutos regionais apresentaram até leve aumento, embora sem significado estatístico (Tabela 2).

Nas três edições da POF, domicílios com menor renda *per capita* tenderam à maior participação calórica relativa dos grupos de peixes de água doce, de macaxeira e derivados, e do total de alimentos regionais. No entanto, não houve relação entre frutos regionais e rendimentos em nenhum momento (Tabela 3).

**Tabela 3:** Média (IC95%) da participação calórica relativa da disponibilidade domiciliar de alimentos regionais no Amazonas por quarto de renda per capita nas POFs 2002–2003, 2008–2009 e 2017–2018.

Quarto de renda domiciliar per capita	2002–2003	2008–2009	2017–2018
Macaxeira e derivados			
1º quarto	21,60 (18,80–24,40)	16,81 (14,23–19,40)	3,22 (1,93–4,51)^b^
2º quarto	15,91 (13,25–18,58)	12,90 (11,08–14,71)	3,40 (2,48–4,32)^b^
3º quarto	11,01 (8,93–13,08)	11,88 (9,82–13,94)	2,68 (1,77–3,60)^b^
4º quarto	7,15 (5,52–8,77)	10,02 (8,37–11,67)	3,01 (1,90–4,13)^b^
Peixes de água doce			
1º quarto	8,97 (7,22–10,71)	6,24 (4,38–8,10)	4,32 (1,89–6,74)
2º quarto	8,25 (6,06–10,44)	4,90 (3,83–5,97)^a^	3,08 (1,89–4,27)
3º quarto	6,74 (4,73 – ,75)	4,27 (3,30–5,25)	2,24 (1,35–3,14)^b^
4º quarto	4,79 (3,11–6,47)	4,19 (3,28–5,11)	2,13 (1,04–3,21)^b^
Frutos regionais			
1º quarto	0,74 (0,34–1,14)	0,58 (0,30–0,87)	0,62 (0,28–0,97)
2º quarto	1,56 (0,47–2,66)	0,76 (0,44–1,07)	0,19 (0,10–0,28)^b^
3º quarto	0,45 (0,16–0,73)	0,47 (0,13–0,81)	0,41 (0,09–0,74)
4º quarto	1,03 (0,11–1,96)	0,34 (0,19–0,50)	0,75 (0,38–1,12)
Total de alimentos regionais			
1º quarto	31,31 (28,00–34,62)	23,64 (19,80–27,48)^a^	8,16 (5,03–11,29)^b^
2º quarto	25,73 (22,49–28,98)	18,56 (16,42–20,71)^a^	6,67 (5,13–8,22)^b^
3º quarto	18,19 (15,29–21,09)	16,75 (14,08–19,43)	5,34 (4,08–6,60)^b^
4º quarto	12,97 (10,52–15,42)	14,62 (12,67–16,57)	5,89 (4,24–7,54)^b^

^a^ diferença estatisticamente significativa entre a POF 2002–2003 e a POF 2008–2009.

^b^ diferença estatisticamente significativa entre a POF 2008–2009 e a POF 2017–2018.

No grupo de macaxeira e derivados, houve redução significativa em todas as classes de renda, porém somente de 2008–2009 para 2017–2018. No grupo dos peixes de água doce, no primeiro intervalo entre as pesquisas, foi observado declínio significativo somente no 2º quarto, passando de 8,25% (2002–2003) para 4,90% (2008–2009). Já, em 2017-2018, a redução desse grupo atingiu os estratos de renda dos 3º e 4º quartos. No grupo dos frutos regionais, destaca-se a redução da aquisição domiciliar entre 2008–2009 e 2017–2018, apenas do 4º quarto, passando de 0,76% para 0,19%. Para o total de alimentos regionais foi observada redução estatisticamente significativa nos quartos de rendas mais baixos de 2002–2003 para 2008–2009 e, em todos os estratos de renda, no período seguinte (Tabela 3).

Ao analisar a disponibilidade domiciliar do total de alimentos regionais no Amazonas conforme características da pessoa de referência da família, foi observado que, em todos os períodos pesquisados, houve uma tendência de maior participação calórica relativa quando essa pessoa era do sexo masculino, mais velho e com menor escolaridade. Em relação às alterações, de 2002–2003 para 2008–2009 foi possível constatar que a redução foi significativa quando se tratava de pessoa do sexo masculino (de 24,42% para 19,37%), até 39 anos (de 22,19% para 16,61%) e com 0 a 4 anos de escolaridade (de 32,44% para 24,51%). Já as alterações de 2008–2009 para 2017–2018 não seguiram essa tendência, ocorrendo em todos os extratos, independentemente de sexo, faixa etária e escolaridade da pessoa de referência do domicílio (Tabela 4).

**Tabela 4:** Média (IC95%) da participação calórica relativa da disponibilidade domiciliar do total de alimentos regionais do Amazonas segundo características da pessoa de referência do domicílio nas POF 2002–2003, 2008–2009 e 2017–2018.

Variáveis sociodemográficas	2002–-2003	2008–2009	2017–2018
Sexo da pessoa de referência do domicílio			
Feminino	17,34 (14,67–20,01)	16,16 (14,59–17,73)	5,10 (4,06–6,15)^b^
Masculino	24,42 (22,73–26,12)	19,34 (17,07–21,60)^a^	7,47 (5,88–9,06)^b^
Faixa etária da pessoa de referência do domicílio (anos)			
Até 39	22,19 (19,97–24,42)	16,49 (14,47–18,50)^a^	5,09 (3,69–6,48)^b^
40–59	21,57 (19,30–28,83)	17,92 (15,88–19,96)	6,46 (5,13–7,78) ^b^
≥ 60	26,22 (21,33–31,10)	22,73 (19,75–25,71)	9,32 (6,57–12,07)^b^
Escolaridade da pessoa de referência do domicílio (anos)			
0–4	32,44 (29,83–35,05)	24,50 (21,50–27,50)^a^	12,11 (8,17–16,05)^b^
5–8	17,51 (14,99–20,02)	19,34 (16,56–22,12)	6,61 (5,27–7,96)^b^
> 8	12,57 (10,72–14,43)	12,79 (11,46–14,12)	4,87 (3,87–5,87)^b^

^a^ diferença estatisticamente significativa entre a POF 2002–2003 e a POF 2008–2009.

^b^ diferença estatisticamente significativa entre a POF 2008–2009 e a POF 2017–2018.

## DISCUSSÃO

A análise da participação domiciliar dos alimentos regionais no Amazonas ao longo dos 15 anos compreendidos pelas três POFs revelou que um novo cenário vem se configurando no estado. Comparado com o restante do país, fica evidente a expressividade que os alimentos regionais já tiveram na alimentação local. Entretanto, os resultados indicam que o Amazonas parece estar passando por perdas de suas características alimentares típicas, que justamente o diferenciavam do contexto nacional. De modo semelhante, um estudo recente que também se baseou em dados das POFs identificou baixa disponibilidade domiciliar de alimentos regionais nas macrorregiões do país, indicando perda da regionalidade com tendência de queda desses itens na dieta dos brasileiros, seguida de estagnação, no período entre 2002 e 2018^20^.

Além do possível efeito dessas mudanças na identidade cultural alimentar, que por si só já é preocupante, outros aspectos merecem reflexão em relação ao Amazonas. Boa parte das calorias disponíveis nos domicílios, principalmente nos estratos rurais e de menores rendas, eram preenchidas pelos grupos de macaxeira e derivados e peixes de água doce, alimentos classificados como *in natura* ou minimamente processados. Este perfil correspondia ao recomendado pelo Guia alimentar para a população brasileira, que baseia a alimentação em alimentos frescos e em preparações culinárias^21^. Entretanto, à medida que tais grupos de alimentos regionais tiveram a aquisição reduzida, as substituições podem não estar ocorrendo de forma adequada, afetando, assim a qualidade da alimentação, pois tem se reproduzido também na Região Norte a tendência nacional de perda progressiva de espaço dos alimentos *in natura* ou minimamente processados, e de ingredientes culinários para os alimentos processados e ultraprocessados^22^.

Ademais, a macaxeira e seus derivados fazem parte do cotidiano alimentar local, especialmente na forma de farinha de mandioca, considerada um alimento tradicional da região amazônica, herdado da cultura dos índios nativos^9^. A farinha de mandioca representa importante fonte de energia e carboidratos complexos. Seu conteúdo de fibras por 100g do produto (6,5g) é superior ao do arroz polido (1,6g), e até mesmo ao do arroz integral (2,8g), contendo, ainda, quantidades apreciáveis de piridoxina, manganês, magnésio, ferro, cálcio e zinco^18^. A farinha de mandioca também tem sido explorada como um promissor alimento funcional, podendo apresentar benefícios fisiológicos devido ao seu conteúdo em fibras e amido resistente^23^

O peixe, por sua vez, é um dos recursos naturais mais abundantes na região, sendo seu decréscimo na aquisição domiciliar duplamente negativo. Primeiro, porque o peixe possui um significado para a população amazonense que extrapola a questão alimentar, apresentando extrema relevância cultural e socioeconômica^24,25^. E, segundo, pela reconhecida qualidade nutricional desse alimento. Inúmeras evidências científicas demonstram que o peixe é uma excelente alternativa para adequação da ingestão de proteínas, em função da elevada quantidade de ácidos graxos essenciais e micronutrientes, sendo consensual recomendar seu consumo regular como parte de uma dieta equilibrada^26^.

Em relação à redução da participação dos alimentos regionais no meio rural, existe uma compreensão de que a sazonalidade dos rios (períodos de enchente, cheia, vazante e seca) interfere diretamente na atividade pesqueira e na produção local de alimentos. Logo, em determinadas épocas do ano é comum ocorrer a busca por fontes alternativas de nutrientes. Entretanto, na última década foi registrada ocorrência de maiores enchentes (2009, 2012 e 2015) e grandes secas (2005, 2010 e 2016), atribuídas às mudanças climáticas mais recentes^27^. Tais oscilações nos regimes dos rios têm obrigado a população a realizar adaptações cotidianas, que, aliadas à vulnerabilidade financeira e à busca por uma modernidade alimentar, podem estar ocasionando a incorporação, de modo definitivo, de novos itens aos hábitos alimentares das famílias.

A análise qualitativa das transformações na rede urbana no Alto Solimões (sudoeste do Amazonas) pela perspectiva da alimentação observou marcante presença do frango industrializado congelado nos municípios estudados, devido, em parte, às questões econômicas^28^.

Pesquisa realizada com famílias de baixa renda da Região Metropolitana de Manaus também observou que, apesar do pescado ser a fonte de proteína animal preferida dos entrevistados (41%), o frango era o alimento mais frequente em suas mesas (69%), mesmo com a baixa aceitabilidade deste (3%), sendo sugerido que tais escolhas seriam consequências da limitação de renda^29^. De fato, o estudo que buscou avaliar o panorama do consumo de peixes pela população brasileira identificou a região Norte como a única em que a população possui preferência por peixes em suas refeições^30^. Apesar disso, em 2008-2009 o frango passou a responder por 7,1% das calorias disponíveis nos domicílios do estado, superando o peixe e passando a ser a principal fonte de proteínas do cardápio amazonense^7^.

Diante disso, torna-se importante assegurar o acesso a alimentos em qualidade e quantidade adequadas, baseado em práticas promotoras da saúde, que respeitem a biodiversidade. As dimensões econômicas e biológicas da produção, comercialização e utilização dos alimentos devem estar articuladas à sustentabilidade socioambiental^1^.

A pesca é um dos segmentos do setor primário que mais produz empregos no Amazonas, gerando renda a um contingente de diferentes níveis socioeconômicos. É particularmente fundamental às populações que residem às margens dos rios, pois representa uma forma de fixar essas pessoas em seu local de origem. Todavia, a pesca na região precisa ainda aperfeiçoar os processos de manejo, beneficiamento, conservação, gerenciamento de resíduos, comercialização e logística^30^.

Da mesma forma, o cultivo da mandioca no estado envolve essencialmente produção artesanal e familiar. Constitui numa atividade que requer pouco investimento e processamento simples para obtenção dos seus subprodutos, farinhas e féculas, que são de fácil conservação e comercialização. Iniciativas locais recentes têm buscado agregar valor à farinha de mandioca, garantindo sua origem, respeito ao meio ambiente e às populações tradicionais, sendo essa uma forma de valorizar o produto regional e estimular seu consumo^31^.

Contudo, apesar do forte incentivo para sua produção, há ainda a necessidade de investimentos em pesquisa e gestão adequada da atividade farinheira, de modo a promover um maior controle da qualidade, profissionalizar a mão-de-obra e aumentar a competitividade do setor, estimulando, assim, as famílias que dela dependem para seu sustento. Portanto, a atividades pesqueira e a produção de farinha de mandioca no Amazonas requerem maior atenção dos órgãos oficiais, para que sejam conduzidas com responsabilidade social e ambiental, contribuindo, assim, para a segurança alimentar da região.

Uma limitação do estudo seria não computar o consumo de alimentos fora do domicílio. Contudo, a região Norte tem apresentado os menores percentuais de despesas com alimentação neste contexto: 19,1% em 2002-2003, 21,4% em 2008-2008, e se manteve nesse mesmo patamar em 2017-2018, contra 24,1%, 31,1% e 32,8% no Brasil, respectivamente, nos três períodos da POF^16^. Além disso, pesquisa sobre o consumo de alimentos fora do domicílio demonstrou que essa prática tem sido mais prevalente na área urbana, entre os mais jovens e com maior renda, diferente do presente estudo, cuja maior redução dos alimentos regionais foi encontrada na área rural e entre as menores faixas de renda^32^.

Por fim, é possível que parte das diferenças observadas entre os grupos possa ter sido em função da sazonalidade. As unidades primárias de amostragem foram distribuídas aleatoriamente entre os quatro trimestres do ano, assegurando que nestes os estratos econômicos estejam representados pelos domicílios selecionados. Isto, contudo, não necessariamente garante que os grupos, especialmente de idade e sexo, estejam igualmente distribuídos entre os trimestres. Nesta situação, a comparação entre estas categorias estaria parcialmente distorcida.

Como ponto forte do estudo, destaca-se a análise pioneira dos dados da POF, com ênfase na aquisição de alimentos típicos de um estado. Considerando os diversos contextos alimentares, socioeconômicos e culturais que o Brasil possui, torna-se relevante analisar se outras localidades também não estão vivenciando fenômeno semelhante, de redução da participação de itens regionais da dieta. Identificar alimentos tradicionais que necessitam de incentivo na produção, na comercialização e no consumo pode constituir um estágio inicial relevante no processo de promoção da alimentação saudável de forma contextualizada com a cultura local.

A análise da evolução da disponibilidade domiciliar de alimentos regionais no Amazonas, estimada com base nas POFs 2002-2003, 2008-2009 e 2017-2018, possibilitou o entendimento de suas características alimentares próprias, sugerindo que a transição alimentar em nível local pode não estar ocorrendo da mesma forma que no cenário nacional. Houve redução significativa da presença de alimentos regionais nos três períodos analisados, atingindo principalmente os domicílios da zona rural e com menor renda, bem como as famílias cuja pessoa de referência era do sexo masculino, mais jovem e com menor escolaridade.
